# An Algorithm for Automatic Rib Fracture Recognition Combined with nnU-Net and DenseNet

**DOI:** 10.1155/2022/5841451

**Published:** 2022-02-25

**Authors:** Junzhong Zhang, Zhiwei Li, Shixing Yan, Hui Cao, Jing Liu, Dejian Wei

**Affiliations:** ^1^School of Clinical, Shandong University of Traditional Chinese Medicine, Jinan 250355, China; ^2^School of Intelligence and Information Engineering, Shandong University of Traditional Chinese Medicine, Jinan 250355, China; ^3^Shanghai Daosh Medical Technology, Shanghai 201203, China

## Abstract

Rib fracture is the most common thoracic clinical trauma. Most patients have multiple different types of rib fracture regions, so accurate and rapid identification of all trauma regions is crucial for the treatment of rib fracture patients. In this study, a two-stage rib fracture recognition model based on nnU-Net is proposed. First, a deep learning segmentation model is trained to generate candidate rib fracture regions, and then, a deep learning classification model is trained in the second stage to classify the segmented local fracture regions according to the candidate fracture regions generated in the first stage to determine whether they are fractures or not. The results show that the two-stage deep learning model proposed in this study improves the accuracy of rib fracture recognition and reduces the false-positive and false-negative rates of rib fracture detection, which can better assist doctors in fracture region recognition.

## 1. Introduction

Rib fracture is a common clinical trauma of the chest, specifically a complete or partial break in the continuity of the rib structure. Rib fractures can be caused by a variety of reasons, such as falls, traffic accidents, and fights. They are more common not only for children and the elderly but also for young and middle-aged people [1]. Most patients with rib fractures have more than one fracture area, so it is important to detect all areas of trauma in a short time for follow-up treatment [2]. Computed tomography (CT) is an important medical aid used to diagnose rib fractures in the chest [3]. However, each patient's chest CT image consists of hundreds of slices [4], which is time-consuming and labor-intensive to manually review. It not only increases the workload of the orthopedic medical staff but also easily leads to visual and psychological fatigue, which will increase the probability of misdiagnosis or even missed diagnosis.

Existing systems for the diagnosis of rib fractures can be broadly classified into two categories [5]. The first category is traditional fracture recognition models, which are used to obtain suspected fracture areas and assist the physician in diagnosis [6]. The second type is fracture recognition models based on deep learning [7]. The following are characteristics that exist in the current rib fracture diagnosis using deep learning: (1) the CT image-based rib fracture dataset has samples with doubtful annotation [8], and different doctors have different annotations for the same case [9]. The doubtful annotation is a great challenge for deep learning models. (2) In general, CT images of fractures are 3D medical images. Deep learning models dealing with 3D data usually have problems such as occupying large memory, slow computation speed, and being prone to overfitting [10]. (3) Deep learning-based fracture region detection models usually suffer from high false-negative and false-positive rates.

To solve the above problems, we propose a rib fracture region recognition model based on the nnU-Net [11] segmentation network and DenseNet [12] classification network, which consists of two stages of training. In the first stage, segmentation of rib fracture regions is completed to generate candidates of fracture regions. At the second stage, the secondary judgment of candidate fracture regions is completed. DenseNet classification network is mainly performed to remove false-positive fracture regions. The experiments show that the new proposed model improves the accuracy of rib fracture recognition by achieving a 95% recognition rate for rib fractures and reducing the false-positive and false-negative rates for rib fracture detection to 5%. The rib fracture recognition algorithm proposed in this study can well assist physicians in the recognition of all fracture regions.

## 2. Related Work

In recent years, the rapid development of deep learning has made a great contribution to medical image-assisted diagnosis. Many researchers have applied deep learning techniques to fracture-assisted diagnosis. It has been proven that the use of fracture-assisted diagnosis systems can improve the accuracy of the doctor's recognition of fractures and effectively save time. For example, Tourassi et al. applied deep convolutional networks (ConvNets) to automatically detect posterior spine fractures. They used the multi-atlantoaxial fusion technique to segment spine and its posterior vertebrae in spine CT and predict the probability of fracture at the image edges using ConvNets (three orthogonal patches in axial, coronal, and sagittal planes) in a 2.5D manner [13]. This method is effective in improving the sensitivity of posterior spine fracture identification. Olczak et al. selected five openly available deep learning networks and trained them to determine fracture, lateral body, and examination views for 256,000 wrist, hand, and ankle x-rays. The experimental results showed that all networks achieved over 90% accuracy in identifying lateral body parts and examination views [14]. Lindsey et al. developed a deep convolutional neural network (DCNN) to assist emergency medicine clinicians in reading x-rays of fracture patients, and experimental results showed that the average misinterpretation rate of emergency medicine clinicians was relatively reduced by 47% with the assistance of this system [7]. Raghavendra et al. proposed an automated technique for thoracolumbar fracture detection based on convolutional neural networks (CNNs) [15], which was able to perform thoracolumbar fracture detection without segmenting the vertebral body, and its detection accuracy was able to reach 99.1% [16]. Takaaki et al. experimentally compared the intertrochanteric fracture diagnostic performance of convolutional neural networks and orthopedic surgeons through the radiograph of proximal femoral [17]. The study showed that convolutional neural networks were three percentage points more accurate than orthopedic surgeons in detecting intertrochanteric fractures. Pranata et al. evaluated the performance of the residual network (ResNet) and visual geometry group (VGG) for heel fracture detection and used the classification results of the better-performing ResNet as input to the SURF algorithm for detecting fracture location and type [18], which validated the feasibility of deep learning neural networks for automatic heel fracture detection.

In previous studies, numerous researchers have also applied deep learning to the detection of rib fractures. To effectively detect and segment rib fracture regions, Jin et al. proposed a deep learning model named FracNet [19], which was based on the classical segmentation network-3D UNet, improved by a sampling strategy during training, and did not rely on the extraction of the rib centerline. This method achieved a detection sensitivity of 92.9% and reduced the time required for clinical testing. Weikert et al. evaluated the diagnostic performance of automatic detection of acute and chronic rib fractures using a deep learning algorithm for whole-body trauma CT [20]. The algorithm consisted of a ResNet-based region proposal phase followed by a fast region-based CNN, which had a final sensitivity of 87.4% and a specificity of 91.5% for rib fracture detection. Zhou et al. evaluated the performance of a two-dimensional convolutional neural network (CNN) model to automatically detect and classify rib fractures and was able to output a structured report [21]. After using this rib fracture automatic detection system to aid clinical trials, it was found that the diagnostic accuracy of radiologists increased from 80.3% to 91.1%, sensitivity increased from 62.4% to 86.3%, and significantly reduced the time required for diagnosis. Meng et al. proposed a heterogeneous neural network consisting of a cascaded feature pyramid network and a classification network for rib fracture detection and classification. They compared the effectiveness of CT images with and without a deep learning model for rib fracture detection and classification [22]. The experimental results show that, with the aid of the deep learning model, clinicians can effectively improve the recall rate and classification accuracy of CT images of rib fractures. Castro-Zunti et al. evaluated the performance of InceptionV3 [23], ResNet50 [24], MobileNetV2 [25], and VGG16 [26] models when classifying acute, aged, and nonfractured ribs in axial CT images [27]. The experimental results showed that the model consisting of the first seven blocks of InceptionV3 was more accurate and faster and achieved a 5-fold cross-validated accuracy and macrosensitivity of 96% and 94%, respectively. These preliminary works provide us with feasible methods for studying rib fracture recognition, but most of the studies summarized above were conducted on a two-dimensional basis, losing three-dimensional information. Therefore, we propose an algorithm for automatic rib fracture recognition with nnU-Net and DenseNet in this study.

## 3. Materials and Methods

### 3.1. Rib Fracture Dataset

**Figure 1:**
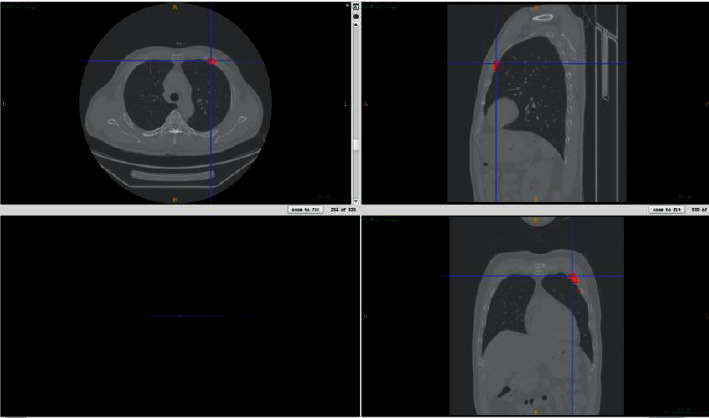
Example of a 3D rib CT image.

The experimental data were the publicly available RibFrac Dataset from the MICCAI 2020 RibFrac Challenge: Rib Fracture Detection and Classification competition, which was published by Liang Jin et al., the authors of the FracNet network structure. The data can be accessed at https://ribfrac.grand-challenge.org/dataset/. The competition dataset contains a total of 420 samples from the training set, 80 samples from the validation set, and 160 samples from the test set. The data are 3D rib CT images annotated by a number of radiologists with different years of experience in the interpretation of chest CT. An example image of a 3D rib CT data is shown in Figure 1. The lower left view shows the current 3D view, and the remaining three views show the results of slicing the data in the axial, coronal, and sagittal planes, respectively. The blue crosshairs represent the current slice position, and the red area is the rib fracture region that was manually marked by physicians.

### 3.2. Dataset Preprocessing

Considering that the test set samples that were not annotated could not be used for calculating prediction accuracy, 160 test set samples were removed from this experiment. Due to the limitation of the experimental equipment and GPU computing power, it was impossible to use the whole training set for training. So, we chose 200 training samples with 1,910 different fracture regions, which can reflect the proportional characteristics of the original dataset, as the training set data. The validation set of the dataset contains no rib fracture images. To reduce the model validation time, this experiment removes no rib fracture images and uses the 3D CT images of 60 rib fractures in the validation set.  Table 1 shows the statistics of rib fracture regions.

From Table 1, after integrating the fracture labels, the ratio of the number of fracture regions in the training and validation sets is approximately 8 : 2, which is in line with the common ratio of data in the training and validation sets.

## 4. Model Description

**Figure 2:**
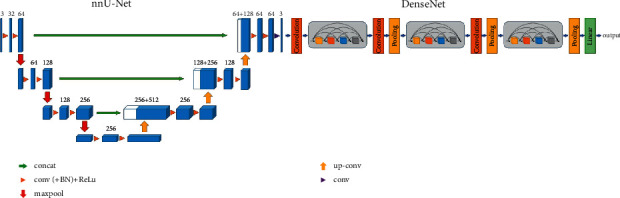
The network structure model proposed in this study. It consists of nnU-Net and DenseNet.

The structure of our model includes the rib fracture region segmentation and the fracture region false-positive exclusion. The first stage used nnU-Net as the fracture region segmentation model, and the second stage used DenseNet as the fracture classification model. Since the second stage of the fracture region false-positive exclusion experiment was based on the first stage of the fracture region segmentation experiment, the network structure of the two-stage rib fracture automatic recognition model based on deep learning proposed in this study is shown in Figure 2.

### 4.1. Stage1: Regional Segmentation of Rib Fractures

This stage mainly completes the segmentation of the rib fracture region and generates the fracture region to be candidates. The main steps are as follows: a series of data preprocessing is taken on the 3D CT rib fracture training set. Then, nnU-Net is selected as the segmentation model and the processed training set is fed into this model for segmentation model training. After training, the rib images in the validation set are predicted to be segmented to generate the fracture regions to be candidates, which facilitate further determination of false positives in the segmented regions.

#### 4.1.1. Preprocessing

(a) Category label processing: the fracture region of this competition dataset was labeled using instance segmentation, with the original label containing five values. 0 indicates the background region, 1–4 represents different types of rib fractures, respectively, and −1 denotes that this region is a rib fracture. Since the images are blurred and ambiguous, it makes rib fracture difficult to be diagnosed and no specific category can be given. In practice, all labels 1–4 and −1 are combined into one category to increase the number of images of rib fracture regions, reducing the influence of suspected fracture regions in the sample on the segmentation results and increasing the accuracy of rib fracture segmentation. Therefore, this experiment is binary fracture region segmentation regardless of the category of fracture.
(b) Since there are inconsistencies in the resolution of the 3D CT fracture images in the training set, they need to be adjusted to a uniform resolution, e.g., 1 mm × 1 mm × 1 mm voxel size, which varies with the training set. The result is obtained by calculating the average voxel size of the training set. After determining the voxel size, each sample in the training set is resampled to obtain the new image size with the following equation.
  (1) $$\begin{array}{c} \text{size}=\text{spacing}\times \text{voxel}. \end{array}$$
In equation (1), *size* is the new 3D image size, spacing is the calculated voxel size, and voxel is the 3D pixel value.
(c) By counting the range of HU (Hounsfield unit) values for pixels within the mask of the entire dataset, a range of HU values in the percentage range of [0.5, 99.5] was cropped and then normalized using the z-score method. In particular, each voxel value of each 3D CT sample is normalized to a mean of 0 so that the processed 3D CT data conforms to a standard normal distribution, i.e., with a mean of 0 and a standard deviation of 1. Such processing facilitates model training and model convergence, improving the training speed of the model with the following equation:
  (2) $$\begin{array}{c} y=\frac{x-\text{mean}}{\text{std}}. \end{array}$$
In equation (2), *y* is the normalized data, mean denotes the mean of the 3D CT sample, and std denotes the variance of the 3D CT sample.

#### 4.1.2. Loss Function

The Dice similarity coefficient is an important indicator for evaluating the degree of overlap between the two samples and the effectiveness of the segmentation, so the segmentation loss function of the model also uses Dice as the loss function. The formula for the Dice loss function is as follows:

(3) $$\begin{array}{c} L_{\text{dice}}=1-\frac{2\left|P\cap T\right|}{\left|P\right|+\left|T\right|}, \end{array}$$

where *P* and *T* are the predicted segmentation mask and the true segmentation annotation, respectively.

When using Dice loss, generally positive samples for small targets will produce severe oscillations. Because in the case of only foreground and background, once some of the pixels in the small target are incorrectly predicted, it will lead to a drastic change in the loss value and the gradient. The loss function is improved, and the final loss function is shown as follows:

(4) $$\begin{array}{c} L=L_{\text{dice}}+L_{\text{ce}}+L_{\text{contour}}, \end{array}$$

where *L*_ce_ denotes the error loss arising from binary cross-entropy. *L*_contour_ denotes the error loss in the 3D profile of the fracture, which is formulated as follows:

(5) $$\begin{array}{c} L_{\text{contour}}={\int^{\;}}_{\Omega }\left|\nabla H_{\varepsilon }\left(\phi \right)\right|, \end{array}$$

where Ω represents the region to be segmented that belongs to the whole sample, *ϕ* represents the level set function, the zero-level curve represents the segmentation boundary, and *H*_*ε*_ represents the smoothed approximation of the Heaviside function.

#### 4.1.3. Model Architecture

In this phase, the nnU-Net is used as the experimental framework. It is a robust adaptive framework based on 2D Unet [28] and 3D Unet [29] that adapts to any medical image dataset and performs different data preprocessing for different datasets. The framework focuses on the following: preprocessing (resampling and normalization), training (loss, optimizer settings, and data augmentation), inference (patch-based strategies, test-time-augmentation integration, and model integration), and postprocessing (e.g., enhanced single-connected domains), while making substantial modifications to the original Unet structure and not adopting new structures such as residual connectivity, dense connectivity, and attention mechanisms.

The framework has three basic versions of the Unet model: 2D Unet, 3D Unet, and 3D cascade Unet. The first two models are 2D Unet and 3D Unet. The third model is a cascaded Unet structure, where the first stage performs coarse segmentation of downsampled low-resolution images, and the second stage combines the results of the first stage for fine-tuning. 3D Unet is used for both stages. Compared with the original Unet, the Unet model in nnU-Net replaces ReLU with leaky ReLU and replaces Batch Norm with Instance Norm.

#### 4.1.4. Postprocessing

After training the segmentation model, the 3D CT images are predicted by means of patches. There will be overlap regions in the sliding prediction, and they will be predicted several times. In this study, we take the maximum value to fuse the prediction results multiple times, as shown in the following equation:

(6) $$\begin{array}{c} P_{s}\left(\text{voxel}\right)=\max\left(P_{s1},P_{s2},\ldots ,P_{si}\right), \end{array}$$

where *P*_*s*_(voxel) represents the segmentation prediction for each voxel and *P*_*si*_ represents the *i-th* segmentation prediction for that voxel.

### 4.2. Stage2: Fracture Area False-Positive Exclusion

In the first stage of the segmentation network, because of the restricted size of the network structure and the insufficient abundance of random negative samples, the variability of morphological features of these false-positive regions and the morphological features of the fracture are not sufficiently learned. A false-positive exclusion network needs to be designed for targeted learning to reduce the false-positive rate. Therefore, the second stage completes the false-positive exclusion of the fracture area and makes a secondary judgment of the predicted fracture area from the first stage.

The main steps are shown as follows. First, a series of data preprocessing is performed on candidate fracture regions produced by segmentation in the first stage. Subsequently, the processed data are fed into the classification model for training a classification model. Eventually, local fracture regions in the validation set are predicted and category labels of the regions are output, with 0 indicating no fracture and 1 indicating a fracture. In addition, the input data for the second stage were preprocessed in the same way as the first stage, and the 3D DenseNet was used as the fracture classification model for the experiments.

#### 4.2.1. Model Inputs

To make the classification model to pay more attention to the contextual information around the fracture region and improve the accuracy of fracture classification, when preprocessing for clipping local fracture regions, three different sizes of local fracture regions are clipped in turn, e.g., 48 × 48 × 48, 64 × 64 × 64, and 80 × 80 × 80. They can also be adjusted according to the actual fracture region size. After cropping local fracture regions of different sizes, three different sizes of fracture images were used as input to train the classification models for each of the three different input sizes.

#### 4.2.2. Postprocessing of Classified Probability Values

After separately training the classification model for the three different input sizes, the final classification results are performed using a weighted average, as shown in the following equation:

(7) $$\begin{array}{c} P_{c}=\lambda_{c1}\times P_{c1}+\lambda_{c2}\times P_{c2}+\lambda_{c3}\times P_{c3}, \end{array}$$

where *P*_*c*_ represents the final classification probability value of a 3D CT image, *λ*_*c*1_ denotes the classification accuracy of the classification model on the validation set under the first size, *P*_*c*1_ represents the probability value of the classification model on the validation set under the first size, *λ*_*c*2_ denotes the classification accuracy under the second size, *P*_*c*2_ represents the probability value under the second size, *λ*_*c*3_ denotes the classification accuracy under the third size, and *P*_*c*3_ represents the probability value under the third size. The given equation is used to calculate whether a fracture is present in the input local 3D CT image.

### 4.3. Model Evaluation and Parameter Settings

#### 4.3.1. Evaluation Metrics

In this study, the diagnostic model evaluation metrics use Dice similarity coefficient (Dice), intersection over union (IoU), average symmetric surface distance (ASSD), and Hausdorff distance (HD).

Dice is a similarity measure used to calculate the similarity of two samples. The value of Dice is in the range [0–1], 1 for the best segmentation result and 0 for the worst. The formula is as follows:

(8) $$\begin{array}{c} \text{Dice}\left(P,T\right)=\frac{2\left|P\wedge T\right|}{\left|P\right|+\left|T\right|}, \end{array}$$

where *P* is the predicted segmentation result, and *T* is the labeled segmentation result. The above equation is also equivalent to the following equation:

(9) $$\begin{array}{c} \text{Dice}\left(P,T\right)=\frac{2\text{TP}}{\text{FP}+2\text{TP}+\text{FN}}. \end{array}$$

IoU is also used to calculate the similarity of two samples. It equals to the overlap of the two regions divided by the pooled portion of the two regions, with the following formula:

(10) $$\begin{array}{c} \text{IoU}=\frac{\text{TP}}{\text{FP}+\text{TP}+\text{FN}}. \end{array}$$

ASSD is the average surface distance, which is an evaluation metric in the medical image segmentation competition CHAOS. ASSD is given by the following equation:

(11) $$\begin{array}{c} \text{ASSD}\left(A,B\right)=\frac{1}{\left|S\left(A\right)\right|+\left|S\left(B\right)\right|}\left(\sum_{a\in S\left(A\right)}\underset{b\in S\left(B\right)}{\min}a-b+\sum_{b\in S\left(B\right)}\underset{a\in S\left(A\right)}{\min}b-a\right), \end{array}$$

where *S*(*A*) denotes the surface voxel of set *A*, *S*(*B*) denotes the surface voxel of set *B*, and *a* and *b* denote the voxels in sets *A* and *B*, respectively. ‖·‖ is the distance paradigm between the point sets *A* and *B*, which generally is the Euclidean distance.

The Dice is more sensitive to the internal filling of the mask, while the HD is more sensitive to the segmented boundary. HD is a measure describing the degree of similarity between two sets of points. It is also a definition of the distance between two sets of points. In contrast to ASSD, HD is also known as the maximum surface distance. HD between *A*={*a*_1_,…, *a*_*p*_} and *B*={*b*_1_,…, *b*_*p*_} is defined as

(12) $$\begin{array}{c} H\left(A,B\right)=\max\left(\underset{a\in A}{\max}\left\{\underset{b\in B}{\min}a-b\right\},\underset{b\in B}{\max}\left\{\underset{a\in A}{\min}b-a\right\}\right). \end{array}$$

#### 4.3.2. Parameter Settings

After data preprocessing, the input data size (patch size column), the training batch size, the number of pooling layers, the resolution of the input image, and the average image size for 2D Unet and 3D Unet are shown in Table 2.

## 5. Results

### 5.1. Quantitative Indicator Assessment Results

Among the above metrics, larger is better for both Dice and IoU, and smaller is better for ASSD and HD-95, indicating that the predicted segmentation results are very close to the true segmentation results. HD-95 denotes the value of the Hausdorff distance multiplied by 0.95, with the aim of eliminating the effect of a very small subset of the outliers. The results can be seen from Table 3:

1. The 2D Unet segmentation model trained with 2D fracture images as input has the worst performance in all indicators, because the 2D image segmentation does not consider the three-dimensional structure of 3D fractures and lacks the contextual information of the *Z*-axis expression of the fracture region.
2. Since 3D_lowres Unet has a small image resolution compared to 3D_fullres Unet during training, the input image size after sampling is smaller. It results in a loss of some detailed information in the 3D image, so it is lower than 3D_fullres Unet in all indexes, 0.82 lower in Dice, 1.21 lower in IoU, 9.21 mm higher in ASSD, and 14.9 mm higher in HD-95. Despite the loss of some information, the 3D Unet segmentation model learns contextual and global information about the fracture region. Its result is better than 2D Unet, with 10.93 higher in Dice, 11.06 higher in IoU, 10.39 mm lower in ASSD, and 31.41 mm lower in HD-95.
3. We use 3D_lowres Unet as a first stage in the 3D cascade Unet and fine-tune it with 3D_fullres Unet, which is better than 3D_lowres Unet in all metrics, with 0.27 higher in Dice and 0.43 higher in IoU. However, it decreases in ASSD and HD-95. Because some detailed information is lost in the first stage, in the second stage, the predictions from the first stage are preprocessed again. The two preprocessing processes make the loss of detail information in the 3D fracture region more severe. Moreover, ASSD and HD-95 indicators belong to the distance category and are extremely sensitive to image resolution, resulting in a slightly worse result for ASSD and HD-95. But Dice and IoU work well.

The result in Table 3 shows that using high-fractional images as input to the segmentation model gives optimal segmentation results. Low-fractional images do not achieve optimal segmentation of the fracture region due to the loss of detail.

### 5.2. Model Loss Curves

**Figure 3:**
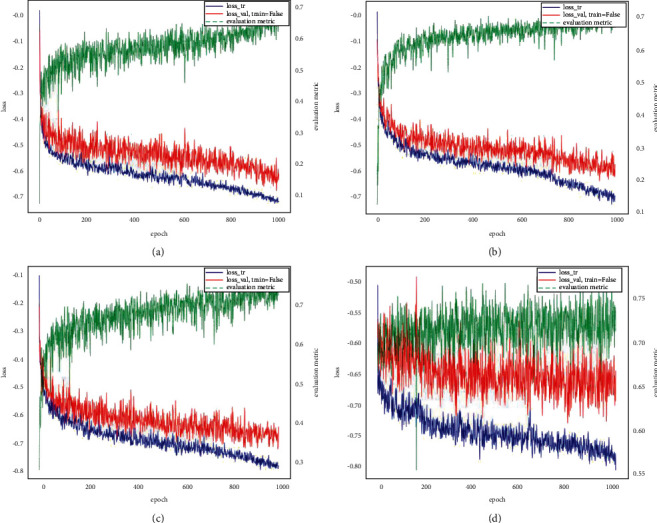
The training set loss curve. (a) Loss curves and Dice value curves for 2D Unet. (b) Loss curves and Dice value curves for 3D_lowres Unet. (c) Loss curves and Dice value curves for 3D_fullres Unet. (d) Loss curves and Dice value curves for 3D cascade Unet.

Figure 3 shows the training set loss curve, the validation set loss curve, and the trained Dice value curve for each Unet model. The following results can be shown in Figure 3:

1. The training loss and validation loss of each Unet model both gradually decrease, indicating that each Unet model slowly converges. The training loss and validation loss of 3D_fullres Unet are the lowest, at around 0.28 and 0.35, respectively.
2. In terms of the training Dice values, it is similar to the results calculated for the quantitative metrics. It also shows that 3D_fullres Unet and 3D cascade Unet have the highest training Dice values, both around 0.75, and 2D Unet has the lowest Dice values, only around 0.68.
3. In the training process of the two-stage 3D cascade Unet, training loss can be seen to significantly drop, while the validation loss shows fluctuations. It is because the two-stage 3D Unet loses some detailed information, which makes the model to learn limited features, and the model gradually shows an overfitting situation.

From the above analysis, 3D Unet performs best in rib fracture region segmentation. Its training results are fed into a false-positive exclusion model for the fracture region in the second phase of the experiment. Its effectiveness in validating the identification of rib fracture regions on the validation set achieved a 95% identification rate and only a 5% false-positive rate. This result indicates that this study can better assist physicians in the identification of rib fracture regions.

## 6. Discussion

We propose a two-stage rib fracture automatic recognition algorithm, which is mainly used to rapidly identify multiple rib fracture lesions in patients with rib fractures, which assists clinicians to diagnose multiple rib fractures from CT scans. As a deep learning-assisted diagnostic system, it was trained on a training set of 1,910 rib fracture regions from 200 patients and tested on a test set of 435 rib fracture regions from 60 patients. It achieved a 5% false-positive rate and 95% recognition rate in the final rib fracture detection and significantly reduced the time required for clinical judgment.

There are two main reasons for these results. First, we used nnU-Net as the network framework for training in the first phase of rib fracture region segmentation. The nnU-Net is proposed as a framework for automatic adaptation to any new dataset. It has a good segmentation result for the RibFrac dataset. Furthermore, we trained each of the three models in the nnU-Net network framework—2D Unet, 3D Unet, and 3D cascade Unet—to select the network model with the best results for subsequent experiments. Second, we conducted an experiment to exclude false-positive fracture regions on the basis of rib fracture region segmentation. We use 3D DenseNet as the classification model. Using the segmented fracture regions as the input to the classification model can narrow the classification range and exclude the false-positive fracture regions in the first stage of segmentation, which effectively improved the accuracy of rib fracture recognition.

From the final identification results, it can be concluded that the two-stage rib fracture automatic recognition algorithm proposed in this study is helpful in assisting physicians in multiple rib fracture recognition and detection, indicating that the artificial intelligence-aided diagnosis system is feasible for multiple rib fracture recognition.

## Figures and Tables

**Table 1:** Regional statistics for rib fractures.

Dataset	Sample size	Number of fracture areas
Training set	200	1910
Validation set	60	435

**Table 2:** Input parameter settings.

Model	Patch size	Batch size	Pooling layers	Spacing/mm	Median size
2D Unet	512 × 512	12	[7, 7]	1.25 × 0.74 × 0.74	328 × 512 × 512
3D_lowres Unet	96 × 160 × 160	2	[4, 5, 5]	2.58 × 1.53 × 1.53	159 × 248 × 248
3D_fullres Unet	96 × 160 × 160	2	[4, 5, 5]	1.25 × 0.74 × 0.74	328 × 512 × 512

**Table 3:** Assessment results of different Unet models with rib fracture segmentation.

Unet	Dice	IOU	ASSD/mm	HD-95/mm
2D	51.05	36.54	35.00	124.42
3D-fuller	62.80	48.81	11.40	78.11
3D-lower	61.98	47.60	20.61	93.01
3D-cascade	62.25	48.03	22.13	100.61

## Data Availability

The experimental data were the publicly available RibFrac Dataset from the MICCAI 2020 RibFrac Challenge: Rib Fracture Detection and Classification competition, which was published by Liang Jin et al., the authors of the FracNet network structure. The data can be accessed at https://ribfrac.grand-challenge.org/dataset/.

## References

[1] Jinliang Y., Guang H. (2016). Strategies, operational techniques and future directions in the treatment of rib fractures. *Journal of Traumatic Surgery*.

[2] Khung S., Masset P., Duhamel A. (2017). Automated 3D rendering of ribs in 110 polytrauma patients: strengths and limitations. *Academic Radiology*.

[3] Jun Y., Guoyu T. (2020). Exploring the working principles of CT in medical imaging technology and new applications. *Imaging Research and Medical Applications*.

[4] Ringl H., Lazar M., Töpker M. (2015). The ribs unfolded - a CT visualization algorithm for fast detection of rib fractures: effect on sensitivity and specificity in trauma patients. *European Radiology*.

[5] He J., Baxter S. L., Xu J., Xu J., Zhou X., Zhang K. (2019). The practical implementation of artificial intelligence technologies in medicine. *Nature Medicine*.

[6] LeCun Y., Bengio Y., Hinton G. (2015). Deep learning. *Nature*.

[7] Lindsey R., Daluiski A., Chopra S. (2018). Deep neural network improves fracture detection by clinicians. *Proceedings of the National Academy of Sciences*.

[8] Urbaneja A., De Verbizier J., Formery A.-S. (2019). Automatic rib cage unfolding with CT cylindrical projection reformat in polytraumatized patients for rib fracture detection and characterization: feasibility and clinical application. *European Journal of Radiology*.

[9] Lenga M., Klinder T., Bürger C., Berg J. V., Franz A., Lorenz C. Deep learning based rib centerline extraction and labeling.

[10] Xu X., Zhou F., Liu B., Fu D., Bai X. (2019). Efficient multiple organ localization in CT image using 3D region proposal network. *IEEE Transactions on Medical Imaging*.

[11] Isensee F., Jaeger P. F., Kohl S. A. A., Petersen J., Maier-Hein K. H. (2021). nnU-Net: a self-configuring method for deep learning-based biomedical image segmentation. *Nature Methods*.

[12] Huang G., Liu Z., Maaten L. V. D., Weinberger K. Q. Densely connected convolutional networks.

[13] Tourassi G. D., Armato S. G., Roth H. R. (2016). Deep convolutional networks for automated detection of posterior-element fractures on spine CT. *Medical Imaging 2016: Computer-Aided Diagnosis*.

[14] Olczak J., Fahlberg N., Maki A. (2017). Artificial intelligence for analyzing orthopedic trauma radiographs. *Acta Orthopaedica*.

[15] Chen X. C. (2014). *Deep Learning Algorithm and Application Research Based on Convolutional Neural Network*.

[16] Raghavendra U., Bhat N. S., Gudigar A., Acharya U. R. (2018). Automated system for the detection of thoracolumbar fractures using a CNN architecture. *Future Generation Computer Systems*.

[17] Takaaki U., Yuki T., Shinichi G. (2018). Detecting intertrochanteric hip fractures with orthopedist-level accuracy using a deep convolutional neural network. *Skeletal Radiology*.

[18] Pranata Y. D., Wang K. C., Wang J. C. (2019). Deep learning and SURF for automated classification and detection of calcaneus fractures in CT images. *Computer Methods and Programs in Biomedicine*.

[19] Jin L., Yang J., Kuang K. (2020). Deep-learning-assisted detection and segmentation of rib fractures from CT scans: development and validation of FracNet. *EBioMedicine*.

[20] Weikert T., Noordtzij L. A., Bremerich J. (2020). Assessment of a deep learning algorithm for the detection of rib fractures on whole-body trauma computed tomography. *Korean Journal of Radiology*.

[21] Zhou Q.-Q., Wang J., Tang W. (2020). Automatic detection and classification of rib fractures on thoracic CT using convolutional neural network: accuracy and feasibility. *Korean Journal of Radiology*.

[22] Meng X. H., Wu D. J., Wang Z. (2021). A fully automated rib fracture detection system on chest CT images and its impact on radiologist performance. *Skeletal Radiology*.

[23] Szegedy C., Vanhoucke V., Ioffe S., Shlens J., Wojna Z. Rethinking the inception architecture for computer vision.

[24] He K., Zhang X., Ren S., Sun J. Deep residual learning for image recognition.

[25] Sandler M., Howard A., Zhu M., Zhmoginov A., Chen L. C. Mobilenetv2: inverted residuals and linear bottlenecks.

[26] Simonyan K., Zisserman A. Very deep convolutional networks for large-scale image recognition.

[27] Castro-Zunti R., Chae K. J., Choi Y., Jin G. Y., Ko S.-b. (2021). Assessing the speed-accuracy trade-offs of popular convolutional neural networks for single-crop rib fracture classification. *Computerized Medical Imaging and Graphics*.

[28] Falk T., Mai D., Bensch R. (2019). U-Net: deep learning for cell counting, detection, and morphometry. *Nature Methods*.

[29] Çiçek Ö., Abdulkadir A., Lienkamp S. S., Brox T., Ronneberger O., Ourselin S., Joskowicz L., Sabuncu M., Unal G., Wells W. 3D U-net: learning dense volumetric segmentation from sparse annotation.

